# Parthenolide Induces ROS-Mediated Apoptosis in Lymphoid Malignancies

**DOI:** 10.3390/ijms24119167

**Published:** 2023-05-23

**Authors:** Joana Jorge, Joana Neves, Raquel Alves, Catarina Geraldes, Ana Cristina Gonçalves, Ana Bela Sarmento-Ribeiro

**Affiliations:** 1 Laboratory of Oncobiology and Hematology (LOH), University Clinic of Hematology, Faculty of Medicine (FMUC), University of Coimbra, 3000-548 Coimbra, Portugal; 2 Coimbra Institute for Clinical and Biomedical Research (iCBR)—Group of Environmental Genetics of Oncobiology (CIMAGO), FMUC, University of Coimbra, 3000-548 Coimbra, Portugal; 3 Center for Innovative Biomedicine and Biotechnology (CIBB), 3004-504 Coimbra, Portugal; 4 Clinical Academic Center of Coimbra (CACC), 3000-061 Coimbra, Portugal; 5 Hematology Service, Centro Hospitalar e Universitário de Coimbra (CHUC), 3000-061 Coimbra, Portugal

**Keywords:** acute lymphoblastic leukemia, multiple myeloma, lymphoma, NF-κB, NF-κB inhibitors, apoptosis, oxidative stress

## Abstract

Lymphoid malignancies are a group of highly heterogeneous diseases frequently associated with constitutive activation of the nuclear factor kappa B (NF-κB) signaling pathway. Parthenolide is a natural compound used to treat migraines and arthritis and found to act as a potent NF-κB signaling inhibitor. This study evaluated in vitro parthenolide efficacy in lymphoid neoplasms. We assessed parthenolide metabolic activity in NCI-H929 (MM), Farage (GCB-DLBCL), Raji (BL), 697 and KOPN-8 (B-ALL), and CEM and MOLT-4 (T-ALL), by resazurin assay. Cell death, cell cycle, mitochondrial membrane potential (ΔΨ_mit_), reactive oxygen species (ROS) and reduced glutathione (GSH) levels, activated caspase-3, FAS-ligand, and phosphorylated NF-κB p65 were evaluated using flow cytometry. *CMYC*, *TP53*, *GPX1*, and *TXRND1* expression levels were assessed using qPCR. Our results showed that parthenolide promoted a metabolic activity decrease in all cell lines in a time-, dose-, and cell-line-dependent manner. The mechanism induced by parthenolide was demonstrated to be cell line dependent. Nonetheless, parthenolide promoted cell death by apoptosis with significant ROS increase (peroxides and superoxide anion) and GSH decrease combined with a ΔΨ_mit_ reduction across all studied cell lines. Despite the need to further understand parthenolide mechanisms, parthenolide should be considered as a possible new therapeutic approach for B- and T-lymphoid malignancies.

## 1. Introduction

Lymphoid neoplasms comprehend a highly heterogeneous group of diseases currently ranked between the 6th and 7th most common cancers worldwide [1]. These hematological neoplasms originate from the neoplastic transformation of B, T, and NK cells at any stage of differentiation and maturation. It is broadly divided into four main categories: Hodgkin lymphomas, precursor lymphoid neoplasms, mature B-cell neoplasms, and mature T- and NK-cell neoplasms [1,2]. Among others, these include multiple myeloma (MM), diffuse large B-cell lymphoma (DLBCL), Burkitt’s lymphoma (BL), and B- and T-cell acute lymphoblastic leukemia (ALL). The therapeutic regimens currently used to treat these diseases have demonstrated to be quite effective; however, a considerable percentage of patients do not respond to treatment, suffer from severe side effects, or end up relapsing [3,4,5].

The nuclear factor kappaB (NF-κB) transcription factor family comprises five mammalian family members, particularly RelA (p65), cREL, RELB, NF-κB1 (p105 and its precursor p50), and NF-κB2 (p100 and its precursor p52). NF-κB dimers are generally sequestered in cytoplasm by their inhibitor IκB family in an inactive complex. Phosphorylation of IκB by IκB kinase (IKK) on the cytosolic domain and subsequent ubiquitination and proteasome degradation of IκB lead to the activation of NF-κB. The activated NF-κB then translocates to the nucleus and induces the transcription of its many target genes, such as *BCL-2*, *BCL-xL*, *CCND1*, *CMYC*, and *TP53*. This transcription factor family is responsible for regulating various biological processes, such as survival, proliferation, and inflammation [6]. Moreover, it plays a crucial role in normal lymphocyte development, survival, and function acquisition, being transiently activated throughout this process [7,8].

Aberrant activation of NF-κB has already been observed in different types of cancer, and lymphoid malignancies in particular rely on the constitutive activation of NF-κB for cell survival and proliferation [9]. Furthermore, NF-κB activation is also associated with chemotherapy resistance in these diseases. For these reasons, NF-κB inhibition represents an attractive therapeutic option for lymphoid malignancies.

**Figure 1:**
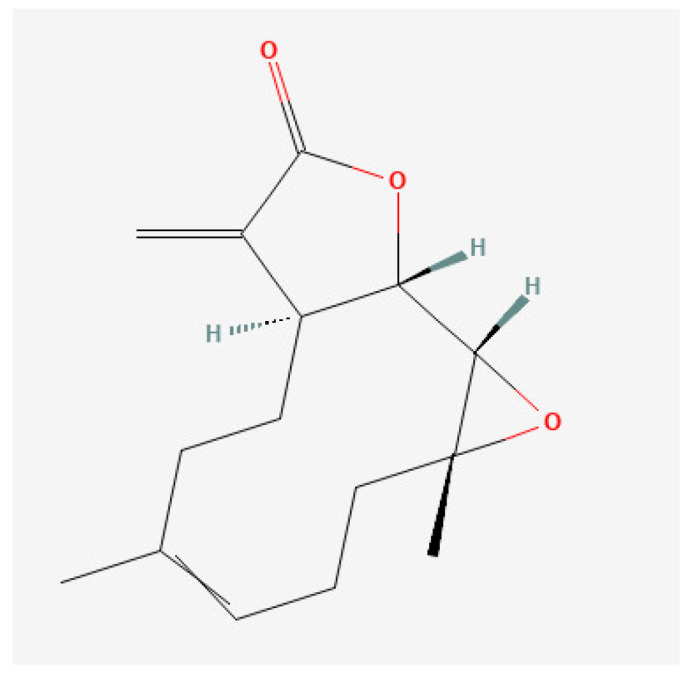
2D molecular structure of parthenolide. PubChem CID 108068.

Parthenolide is a naturally occurring sesquiterpene lactone isolated from *feverfew* (*Tanacetum parthenium*), a traditional medicinal plant that has been conventionally used in the treatment of fever, migraines, and arthritis. Parthenolide has also been highly studied for its anticancer activities in multiple cancer types, including hepatocellular carcinoma, breast cancer, and hematological malignancies [10,11,12,13,14,15,16], among others. The variety of biological activities associated with parthenolide are mainly related to its chemical structure, which includes an α-methylene-γ-lactone ring and epoxide group capable of interacting with the nucleophilic sites of various proteins, ultimately interfering with different signaling pathways (Figure 1) [17]. A series of parthenolide direct targets (p65, IκB kinase, IGF-1, B-RAF, among many others) have already been reported to directly affect several signaling pathways related to tumorigenesis and progression, mainly inhibiting growth, and inducing apoptosis [18,19,20,21]. Moreover, parthenolide-induced apoptosis is also associated with decreased intracellular thiol levels in cancer cells, including free glutathione, while incrementing reactive oxygen species (ROS). Despite its promising results, parthenolide presents high lipophilicity and limited bioavailability. Much research has been concentrated in the development of new alternative delivery strategies, water-soluble synthetic analogs, or even therapeutic combinations that might overcome this limitation [14]. Nonetheless, parthenolide low cytotoxicity together with other attractive features, such as the ability to induce a cytotoxic effect on cancer cells while sparing normal cells and its selective targeting of cancer stem cells (CSC), encourages further research into parthenolide and the anticancer effects of its derivatives on different types of cancers [22,23,24,25].

Our study explored the antitumor effect of parthenolide in several lymphoid neoplastic cell lines, unveiling its role in apoptotic mechanisms and oxidative stress dysregulation.

## 2. Results

### 2.1. Parthenolide Induces a Dose-, Time-, and Cell Line-Dependent Reduction on Lymphoid Neoplastic Cell Line Metabolic Activity

**Figure 2:**
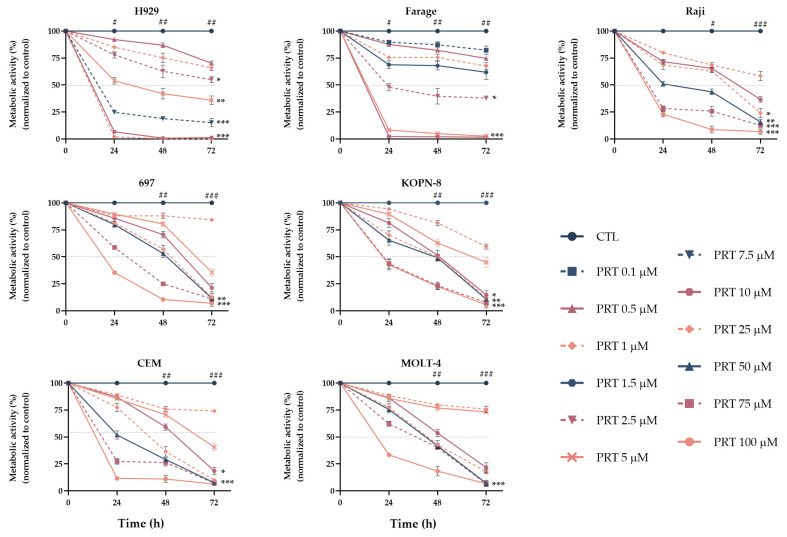
Dose-response curves of parthenolide (PRT) in lymphoid neoplastic cell lines. Cells were incubated with increasing concentrations of parthenolide for 72 h. Results are expressed in percentage (%) normalized to control and represent the mean ± SEM of at least 5 independent experiments. *, p < 0.050; **, *p* < 0.010; ***, *p <* 0.001 compared with untreated cells (CTL); *#, p* < 0.05; ##, *p* < 0.01; ###, *p* < 0.001 compared with 0 h.

To evaluate the potential effect of parthenolide on metabolic activity, seven lymphoid neoplastic cell lines were incubated in the absence and presence of increasing concentrations of parthenolide, ranging between 0.1 µM and 100 µM. Resazurin assay was performed at 24 h, 48 h, and 72 h. Our results showed that parthenolide reduced the metabolic activity in all cell lines in a dose-, time- and cell line-dependent manner, as represented in Figure 2. Studied cell lines presented a half maximal inhibitory concentration (IC_50_) ranging between 1 µM and 10 µM.

To further understand the mechanisms associated with parthenolide in these cell lines, subsequent studies were performed using two concentrations. One concentration was common to all seven cell lines and corresponded with 1 µM; other concentrations closer to the IC_50_ observed for each cell line were 1.5 µM for Farage, 2.5 µM for 697 and KOPN-8, 5 µM for H929 and CEM, 7.5 µM for MOLT-4, and 10 µM for the Raji cell line.

### 2.2. Parthenolide Does Not Induce Cell Cycle Arrest in Lymphoid Neoplastic Cell Lines

Our first approach to evaluate the mechanistic effect of parthenolide was to assess if the decrease in metabolic activity was induced by a cytostatic effect. To evaluate this, cell cycle distribution was assessed using flow cytometry (FC) and a propidium iodide (PI)/RNAse kit. As previously mentioned, two concentrations of parthenolide were used for each cell line for a 72 h incubation. Parthenolide induced cell cycle arrest in a cell line-dependent manner (Table 1). Cell arrest at G_2_/M phase was observed in both the Raji (control: 12.0 ± 1.2; PRT 10 µM: 20.7 ± 1.7; *p* = 0.006) and the MOLT-4 cell lines (control: 3.3 ± 1.3; PRT 7.5 µM: 13.7 ± 0.9; *p* = 0.001). No differences in cell cycle distribution were observed for the other four cell lines: H929, Farage, 697, and CEM.

### 2.3. Parthenolide Induces Caspase-Mediated Cellular Apoptosis in All Lymphoid Neoplastic Cell Lines

**Figure 3:**
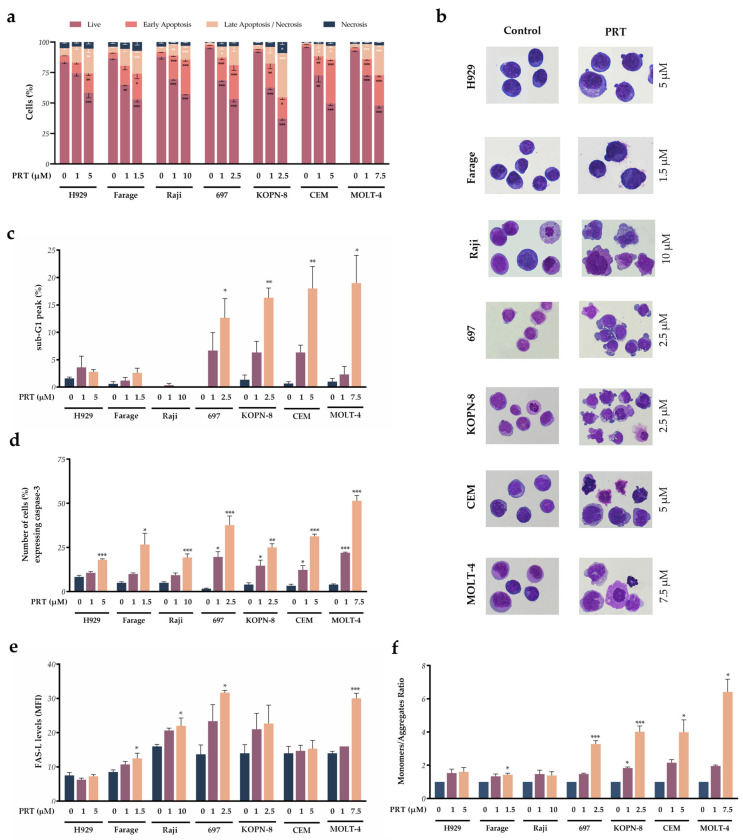
Analysis of cell death induced by parthenolide (PRT) in lymphoid neoplastic cell lines. (**a**) Cell death was evaluated using AV/7-AAD double staining using flow cytometry (FC). Data were expressed in percentage (%) of live, early apoptotic, late apoptotic/necrotic, and necrotic cells. (**b**) Cell morphology was analyzed using light microscopy with May–Grünwald–Giemsa staining (amplification 1000×). (**c**) Sub-G_1_ peak associated with DNA fragmentation identified using a PI/RNAse cell cycle analysis kit. Data were expressed in percentage (%) of cells in Sub-G_1_. (**d**) Activated caspase-3 expression levels as a percentage of (%) of positive cells. (**e**) FAS-L expression levels represented as mean fluorescence intensity (MFI). (**f**) ΔΨ_mit_ measured using JC-1 fluorescent probe by FC. JC-1 probe coexists in monomeric (M) or aggregate (A) forms depending on the mitochondrial potential; M/A ratio represents ΔΨ_mit_ results. Data represent the mean ± SEM of at least 3 independent determinations *, *p* < 0.050; **, *p* < 0.010; ***, *p* < 0.001 compared with untreated cells (control).

To evaluate whether parthenolide also induced a cytotoxic effect, cell death analysis was first assessed using flow cytometry with anexin V (AV) and 7-Aminoactinomycin D (7-AAD) double staining. Our results (Figure 3a) showed that parthenolide induced a dose-dependent increase in early apoptosis and late apoptosis/necrosis with statistical significance in all seven cell lines, with a concomitant decrease in viable cells. The percentage of viable cells was significantly decreased in higher parthenolide concentrations (H929: 58.4 ± 4.1, *p* < 0.001; Farage: 53.0 ± 2.1, *p* < 0.001; Raji: 57.3 ± 0.7, *p* < 0.001; 697: 53.3 ± 2.4, *p* < 0.001, KOPN-8: 37.3 ± 1.3, *p* < 0.001; CEM: 49.7 ± 1.8, *p* < 0.001; MOLT-4: 48.0 ± 2.1, *p* < 0.001). In agreement with these results, morphological analysis performed in these higher concentrations showed typical features of cell death mediated by apoptosis, confirming apoptosis activation by parthenolide. These apoptotic features included nuclear fragmentation and blebbing in the parthenolide-treated cells (Figure 3b). Moreover, DNA fragmentation, another feature of apoptosis, was also observed as a sub-G_1_ peak identified by the same methodology used for cell cycle distribution. This sub-G_1_ peak, as demonstrated in Figure 3c, was increased in all parthenolide incubated conditions except in the Raji cell line. This increase was statistically significant in 697 (control: 0.0 ± 0.0; PRT 2.5 µM: 12.7 ± 3.5; *p* = 0.031), CEM (control: 0.7 ± 0.3; PRT 5 µM: 18.0 ± 4.0; *p* = 0.004) and MOLT-4 (control: 1.0 ± 0.6; PRT 7.5 µM: 19.0 ± 5.0; *p* = 0.010) cell lines.

To further confirm apoptosis induction, we assessed the activation of caspase-3, an effector caspase (Figure 3d), and FAS ligand expression (FAS-L; Figure 3e). Activated caspase-3 expression levels increased with parthenolide incubation in all cell lines compared to the untreated cells. This increase was dose-dependent, being more significant for the higher parthenolide concentrations (Figure 3d). This was more evident in the 697 cell line with a 22.6-fold (37.7 ± 5.2; *p* < 0.001) increase and MOLT-4 with a 12.8-fold (51.5 ± 2.9; *p* < 0.001) increase in caspase-3 expression levels. The H929 and Raji cell lines presented the lowest increase in caspase-3 levels, 2.2- (18.0 ± 0.6; *p* = 0.010) and 3.9-fold (19.3 ± 2.0; *p* < 0.001), respectively.

FAS-L expression levels were also increased in response to parthenolide in all cell lines, except for H929 and CEM cell lines (Figure 3e). The other cell lines presented statistically significant FAS-L levels, at least in the higher concentrations of the compound (Farage—control: 8.5 ± 0.7, PRT 1.5 µM: 12.5 ± 1.5, *p* = 0.047; Raji—control: 16.0 ± 0.6, PRT 10 µM: 22.0 ± 2.3, *p* = 0.044; MOLT-4—control: 14.0 ± 0.6, PRT 7.5 µM: 30.0 ± 1.5, *p* < 0.001), highlighting a possible involvement of the extrinsic apoptotic pathway.

We then evaluated the mitochondrial membrane potential (ΔΨ_mit_) using a JC-1 probe using flow cytometry. Cellular apoptosis is associated with a higher JC-1 monomer (M)/aggregate (A) ratio corresponding to a lower mitochondrial membrane potential. Parthenolide induced a dose-dependent increase in the M/A JC-1 ratio in all the lymphoid neoplastic cell lines, except for the Raji cell line, demonstrating that the intrinsic apoptotic pathway is also involved in parthenolide response (Figure 3f). This increase was particularly substantial in the higher parthenolide concentrations for 697 (3.3 ± 0.2, *p* < 0.001), KOPN-8 (4.0 ± 0.3, *p* < 0.001), CEM (4.0 ± 0.7, *p* = 0.013), and MOLT-4 (6.4 ± 0.7, *p* = 0.013), i.e., the four acute lymphoblastic leukemia cell lines.

### 2.4. Parthenolide Induces Oxidative Stress Imbalance in Lymphoid Malignancies

As parthenolide is also often associated with oxidative stress imbalance, we then assessed the intracellular levels of peroxides and superoxide anion using 2,7-dichlorodihydrofluorescein diacetate (DCFH_2_-DA) and dihydroethidium (DHE) fluorescent probes, respectively, and the intracellular levels of reduced glutathione (GSH), a major antioxidant, using a mercury orange (MO) probe. As demonstrated in Table 2, incubation with parthenolide increased peroxide levels in all seven cell lines with statistical significance in Raji (PRT 10 µM: 107.6 ± 1.6; *p* = 0.030), 697 (PRT 2.5 µM: 142.7 ± 18.4; *p* = 0.028), KOPN-8 (PRT 2.5 µM: 125.5 ± 7.4; *p* = 0.002), CEM (PRT 5 µM: 117.1 ± 1.4; *p* < 0.001), and MOLT-4 (PRT 7.5 µM: 170.9 ± 11.8; *p* < 0.001). Similar results were obtained for superoxide anion levels (Raji—PRT 10 µM: 133.0 ± 9.9, *p* = 0.014; 697—PRT 2.5 µM: 112.3 ± 3.4, *p* = 0.003, KOPN-8—PRT 2.5 µM: 148.3 ± 17.8, *p* = 0.011; CEM—PRT 5 µM: 112.4 ± 1.4, *p* < 0.001; MOLT-4—PRT 7.5 µM: 139.6 ± 7.3, *p* < 0.001).

As an antioxidant defense, GSH levels were significantly reduced in all cell lines when incubated with parthenolide, particularly with the higher doses. This decrease was more pronounced in the Raji cell line (PRT 10 µM: 23.7 ± 1.4, *p* < 0.001). These results further prove that parthenolide effects are mainly mediated by the intrinsic apoptotic pathway.

### 2.5. pNF-κB Suppression by Parthenolide Is Dose- and Cell Line-Dependent

**Figure 4:**
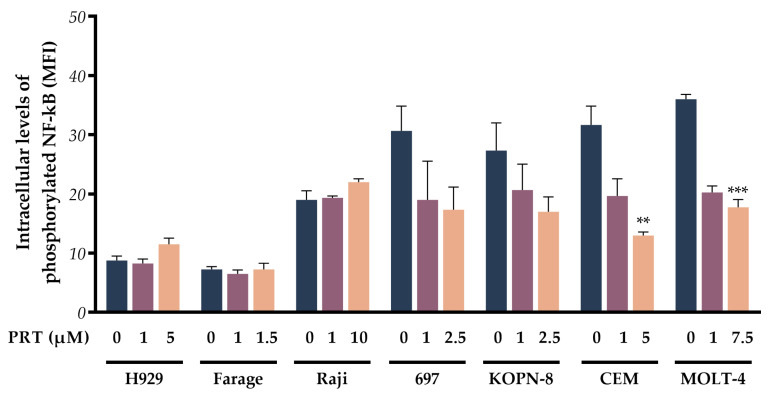
Phosphorylated NF-κB induced by parthenolide in lymphoid neoplastic cell lines. Data are expressed as mean fluorescence intensity (MFI) and represent the mean ± SEM of at least 3 independent determinations **, *p* < 0.010; ***, *p* < 0.001 compared with untreated cells (control).

To assess the inhibition of IKK by PRT, the expression of the active form of NF-kB (p65 subunit) was also evaluated. Parthenolide induced a dose-dependent decrease in pNF-κB expression levels in the four ALL cell lines: 697, KOPN-8, CEM, and MOLT-4 (Figure 4). Among these, T-ALL cell lines presented more expressive results, with a 2.4-fold decrease in CEM cells (*p* = 0.007) incubated with the high concentration of parthenolide and a 2.0-fold reduction in the MOLT-4 cell line (*p* < 0.001), when compared to the control cells. For the other three cell lines (H929, Farage, and Raji), no differences were observed in pNF-κB expression after parthenolide treatment.

### 2.6. Parthenolide Modulates Gene Expression Levels

**Figure 5:**
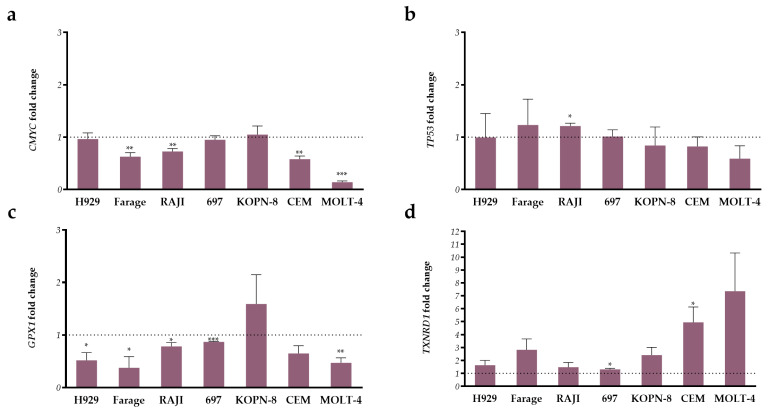
Expression analysis of 5 parthenolide target genes in lymphoid neoplastic cell lines. (**a**) *CMYC*, (**b**) *TP53*, (**c**) *GPX1*, and (**d**) *TXNRD1* gene expression levels. Columns represent the higher parthenolide condition specific for each cell line, and the dotted line represents the expression levels in untreated cells. Data are expressed as mean ± SEM of 3 independent experiments. *, *p* < 0.050; **, *p* < 0.010; ***, *p* < 0.001 compared to the untreated cells (control).

Finally, we measured the expression levels of four genes: two parthenolide target genes, *CMYC* and *TP53*, and two oxidative stress-related genes, *GPX1* and *TXNRD1*, after 72 h incubation with parthenolide at higher doses. As represented in Figure 5, gene expression levels are highly cell line dependent. Parthenolide incubation slightly diminished *CMYC* expression levels in all cell lines, except in the KOPN-8 cell line. However, only the MOLT-4 cell line presented over 50% reduction in CMYC expression (0.1 ± 0.025, *p* < 0.001; Figure 5a). Only the Raji cell line showed statistical differences in *TP53* expression levels (1.2 ± 0.0, *p* = 0.001; Figure 5b).

The oxidative stress-related genes, *GPX1*, which encodes glutathione peroxidase 1, and *TXNRD1*, which encodes for thioredoxin reductase 1, were evaluated. Our results showed that parthenolide significantly decreased *GPX1* expression levels in all cell lines except for CEM (no statistical difference) and KOPN-8, which presented increased levels (Figure 5c). For *TXNRD1* (Figure 5d)*,* all cell lines presented increased expression levels after incubation with parthenolide, with the 697 (1.3 ± 0.07, *p* = 0.013) and CEM (4.9 ± 1.2, *p* = 0.006) cell lines presenting statistical differences when compared to untreated cells.

## 3. Discussion

Several lymphoid cancers highly rely on NF-κB constitutive activation, which is usually mainly related to genetic mutations in this pathway’s key components. Through the induction of anti-apoptotic and proliferative genes, NF-κB activation strongly contributes to tumor promotion and survival. This is particularly important in lymphoid-related neoplasms when it comes to the critical contribution of NF-κB signaling to normal lymphoid development. Therefore, inhibition of NF-κB signaling becomes an attractive target to pharmacologically treat these diseases [9].

Natural compounds have provided a variety of new alternative drugs for cancer treatment. Parthenolide is a phytochemical compound with various biological activities exhibiting anti-inflammatory, antioxidant, and strong antitumor activity. These different activities are mainly associated with its chemical structure, which includes a lactone moiety and epoxide capable of interacting with the nucleophilic sites of numerous proteins interfering with multiple signaling pathways. Apoptosis induction is one of the most important actions of parthenolide, as it is frequently associated with NF-κB inhibition, ROS generation, and mitochondrial dysfunction. This antineoplastic action has already been studied in several solid tumors. However, only a few studies have been performed on hematological neoplasia [17]. One of these studies was performed in primary cell cultures of acute and chronic myelogenous leukemia (AML and CML) patients, also highlighting another important feature of parthenolide: its low cytotoxic effect on normal tissues [22]. Guzman et al. group showed that parthenolide has a selective effect on leukemia stem cells without affecting normal hematopoietic cells, both total and CD34+ cells, thus inducing a small decrease in cell viability [22]. This feature was further demonstrated in other types of normal tissues [23,24,25]. In this study, we evaluated the therapeutic potential of parthenolide in several lymphoid neoplastic cell lines, particularly H929 (multiple myeloma), Farage (germinal center B-cell like (GCB) diffuse large B-cell lymphoma), Raji (Burkitt’s lymphoma), 697 and KOPN-8 (B-acute lymphoblastic leukemia), CEM, and MOLT-4 (T-acute lymphoblastic leukemia). We demonstrated that parthenolide induced a dose-, time- and cell line-dependent metabolic decrease in our cell models. Cell death was significantly increased in all cell lines after parthenolide incubation and is mediated mainly by apoptosis, especially in higher parthenolide doses. This apoptosis induction was primarily confirmed using morphological analysis and identification of a sub-G_1_ peak. We further observed a parthenolide-induced increase in activated caspase-3 levels and a cell-dependent increase in FAS-ligand expression levels. This parthenolide-induced apoptosis was also combined with a significant decrease in the intracellular levels of glutathione, together with the generation of high levels of reactive oxygen levels, namely peroxides and superoxide anions, in all the studied cell lines. Moreover, parthenolide showed induced cell cycle arrest in G_0_/G_1_ in KOPN-8 and G_2_/M in Raji and MOLT-4 cell lines. It also inhibited the expression of intracellular levels of phosphorylated NF-κB p65, particularly in T-ALL, ultimately inhibiting NF-κB activation. Finally, we demonstrated that parthenolide alters the expression levels of *CMYC*, *TP53*, *GPX1*, and *TXNRD1* in a cell line-dependent manner.

Apoptosis induction by parthenolide has been extensively described in several different cancer-associated studies. Recently, a proteomic study conducted by Cui et al. identified several proteins associated with apoptosis promotion in thyroid cancer cells [26]. Despite being highly dependent on the cell line, our results demonstrated a common mechanism associated with apoptosis across the entire cell panel, that is, an increase in ROS with a decrease in GSH, and a reduction in mitochondrial membrane potential. Wen et al. (2002) first demonstrated similar parthenolide-induced apoptosis effects in hepatoma cells, together with activation of caspases (caspases-7, -8, and -9) and overexpression of *GADD153*, a DNA damage-inducible gene. This group further confirmed these results using N-acetyl-l-cysteine (NAC) and buthionine sulfoximine (BSO), a ROS inhibitor and a GSH inhibitor, respectively [27]. ROS induction by parthenolide was also associated with other forms of cell death, depending on the tumor cell type. Autophagy-induced apoptosis was associated with parthenolide treatment in triple-negative breast cancer cells and pancreatic cancer cells by increasing the expression of beclin-1, LC3II, and p62/SQSTM1 [28,29]. A more recent study in hepatocellular carcinoma cells showed that parthenolide depleted intracellular thiols (e.g., GSH) by increasing cytosolic and mitochondrial thiol oxidation, leading to early mitochondrial dysfunction and oxidative stress. This was accompanied by an increase in lipid peroxidation leading to ferroptosis, an irreversible mechanism of cell death [10].

As previously stated, parthenolide mechanisms in our study seemed highly dependent on tumor type. Besides the effects related to apoptosis and oxidative stress which have already been discussed, in the H929 cell line, a multiple myeloma cell model, parthenolide did not alter the phosphorylated NF-κB p65 expression levels. Constitutive activation of NF-κB signaling contributes to MM pathogenesis and is usually related to oncogenic mutations and inflammation [30]. However, despite the NF-κB inhibition described by others in other MM cell models [31], in our study, the parthenolide effect seems to be independent of NF-κB. This might indicate that in our specific MM cell model, activation of NF-κB signaling might occur by the non-canonical pathway rather than the canonical one. Identical results were also observed for the lymphoma cell models of Farage and Raji, also demonstrating a parthenolide effect independent of NF-κB. Only one study evaluated parthenolide treatment in B-lymphoma cells, demonstrating that low expression of BCL-xL, an anti-apoptotic protein, and the NF-κB target gene, sensitizes cells to parthenolide [32]. Moreover, NF-κB constitutive activation in DLBCL is more related to ABC-DLBCL that has a poor prognosis than to GCB-DLBCL, which is the subtype of the Farage cell line and the DLBCL model used in our study [33].

Parthenolide demonstrated apoptosis-associated oxidative stress in B- and T-ALL models and a significant decrease in NF-κB p65 phosphorylation. The first study using parthenolide in B-ALL demonstrated that it induces growth arrest and stress response, specifically in t(4;11) ALL cell lines [34]. Nonetheless, none of our cell lines harbor this specific translocation. However, in KOPN-8 we observed a G_2_/M cell cycle arrest after parthenolide incubation, as well as in the Raji cell line. These are the two less sensitive cell lines, which might indicate a dose-dependent effect of parthenolide. Another important study in ALL using in vitro and in vivo studies demonstrated the potential of parthenolide to inhibit different subpopulations of cells, particularly leukemia-initiating cells, that highly contribute to disease progression and relapse [35]. More recently, a group led by Ede et al. described a parthenolide resistance mechanism proving that bone marrow mesenchymal stem cells (BM-MSCs) release thiols that protect T-ALL cells from parthenolide-induced oxidative stress. The same group also suggested a combination therapy of parthenolide with cystine uptake inhibitors to achieve more significant toxicities in T-ALL [25].

Although it presents a high antitumor potential, parthenolide was shown to be highly lipophilic and to have low solubility, which limits its bioavailability and solubility in blood plasma. For this reason, our and other research groups have been focusing on the development of parthenolide derivatives and of new transport nanoparticles that will possibly increase parthenolide efficacy. For instance, a recently published study in acute myeloid leukemia developed a poly lactide co-glycolide (PLGA)-antiCD44-parthenolide nanoparticle that improved bioavailability and selectively targeted leukemic cells [14].

## 4. Materials and Methods

### 4.1. Cell Lines and Culture Conditions

A panel of seven hematological cell lines was used: NCI-H929 (MM), Farage (GCB-DLBCL), Raji (BL), 697 and KOPN-8 (B-ALL), and CEM and MOLT-4 (T-ALL). All cell lines were maintained in Roswell Park Memorial Institute 1640 (RPMI-1640; Corning, NY, USA), containing 2 mM of L-glutamine, 100 U/mL of penicillin, 100 g/mL of streptomycin (Corning), supplemented with 10% fetal bovine serum (FBS) (Corning). The NCI-H929 cell line was supplemented with 20% FBS, 1 mM sodium pyruvate (Corning), and 50 µM mercaptoethanol (Corning). Cells were maintained at 37 °C in a humidified atmosphere containing 5% CO_2_ and incubated at an optimal density. To ensure mycoplasma-free cultures, cells were periodically tested using PCR.

### 4.2. Metabolic Activity Assay

The metabolic activity of cells incubated with parthenolide was evaluated using resazurin assay. This compound was purchased from APExBIO (Boston, MA, USA) and dissolved in dimethyl sulfoxide (DMSO). All cell lines were incubated for 72 h in the absence and presence of increasing concentrations of parthenolide, ranging between 0.1 and 100 µM. To avoid DMSO cytotoxicity and to ensure consistency between conditions, a 100-fold concentration was used. The cells were plated on a 48-well plate for 72 h. Every 24 h, resazurin was added to a final concentration of 10 mg/mL and then incubated at 37 °C for at least 2 h. The absorbance at 570 nm and 600 nm was measured using a microplate spectrophotometer (SynergyTM HT Multi-Mode Micro-plate Reader, BioTek Instruments, Winooski, VT, USA), and the metabolic activity was calculated as a percentage of control.

### 4.3. Cell Cycle Analysis

The cell cycle distribution was evaluated using FC and a propidium iodide (PI)/RNase cell cycle analysis kit (Immunostep, Salamanca, Spain), as previously described [36]. Briefly, after 72 h of incubation, 1 × 10^6^ of untreated and treated cells were collected and washed with PBS for 5 min at 1000× *g*. The pellet was resuspended in 200 µL of 70% ethanol solution during vortex agitation and incubated for 30 min at 4 °C. Then, cells were washed with PBS and resuspended in 500 µL of PI/RNase solution. Flow cytometry analysis was performed in a FACSCalibur flow cytometer (Becton Dickinson). Results were expressed in the percentage of cells in each cell cycle phase (G_0_/G_1_, S, and G_2_/M) according to PI fluorescence intensity. A sub-G_1_ peak was also identified as apoptotic cells. The cell cycle distribution was analyzed using ModFit^LT^ software V2.0 (Verity Software House, Topsham, ME, USA).

### 4.4. Assessment of Cell Death

To evaluate cell death induced by parthenolide, we used optical microscopy after May–Grünwald–Giemsa staining to study morphological features and flow cytometry (FC), using annexin-V (AV) and 7-Aminoactinomycin D (7-AAD) double staining and a JC-1 probe. FC was also used to evaluate the expression levels of a few apoptotic proteins (FAS ligand (FAS-L) and activated caspase-3) to clarify the mechanisms involved. Cells untreated and treated with two different concentrations of parthenolide were incubated for 72 h and collected for the following measurements.

#### 4.4.1. Annexin V and 7-Aminoactinomycin D Double Staining

Cell death was first evaluated using AV/7-AAD double staining by FC, as described by Lapa, 2020 [37]. Briefly, 0.5 × 10^6^ cells were washed with PBS, centrifuged at 500× *g* for 5 min, resuspended in 100 µL of annexin V binding buffer, and incubated with 2.5 µL of annexin V-APC (Biolegend, San Diego, CA, USA) and 5 µL of 7-AAD (Biolegend) for 15 min in the dark at room temperature (RT). Then, cells were diluted in 300 µL of annexin V binding buffer and analyzed in a FACSCalibur flow cytometer (Becton Dickinson, Franklin Lakes, NJ, USA). At least 25,000 events were acquired using CellQuest software v3.3 (Becton Dickinson) and analyzed using Paint-a-Gate v3.0 (Becton Dickinson). The results were expressed as a percentage of viable cells (AV^−^/7-AAD^−^), initial apoptotic (AV^+^/7-AAD^−^), late apoptotic/necrotic (AV^+^/7-AAD^+^), and necrotic cells (AV^−^/7-AAD^+^).

#### 4.4.2. Morphological Analysis

Optical microscopy was used to assess the morphological features associated with apoptosis and necrosis. A quantity of 1 × 10^6^ cells was collected and seeded in glass slides. Then, smears were stained for 3 min with May–Grünwald solution (Sigma-Aldrich, St. Louis, MO, USA) and for 15 min with Giemsa solution (Sigma-Aldrich). After rinsing with distilled water, cell morphology was analyzed using light microscopy with a Nikon Eclipse 80i microscope equipped with a Nikon digital camera DXm 1200F (Nikon, Tokyo, Japan).

#### 4.4.3. Expression of Apoptosis-Related Proteins using Flow Cytometry

The activated caspase 3 and FAS-L expression levels were assessed using FC with fluorescently labeled monoclonal antibodies. A quantity of 1 × 10^6^ cells was incubated with a monoclonal antibody anti-activated caspase 3-fluorescein isothiocyanate (FITC; BD Pharmingen, Becton Dickinson), and FAS-L (PE; Santa Cruz Technology, Dallas, TX, USA) antibodies according to the manufacturer’s protocol. For intracellular staining of activated caspase 3, cells were fixed with 100 μL of a fix solution (IntraCell, Immunostep, Salamanca, Spain) for 15 min and then washed by centrifugation at 300× *g* for 5 min. Cells were then permeabilized by incubating for 15 min with 100 μL of a permeabilization solution (IntraCell) and the respective antibody. For membrane staining of FASL, cells were incubated with a FAS-L antibody and incubated for 15 min. After washing, cells were analyzed using FC. At least 25,000 events were acquired using CellQuest software (Becton Dickinson) and analyzed using Paint-a-Gate (Becton Dickinson). The results are presented as mean fluorescence intensity (MFI) arbitrary units and represent MFI detected in the cells, which is proportional to the protein concentration in each cell.

#### 4.4.4. Mitochondrial Membrane Potential Assessment

Mitochondrial membrane potential (ΔΨ_mit_) was evaluated using FC with the fluorescent probe JC-1 (Enzo Life Sciences, Farmingdale, NY, USA). After 72 h of incubation in the absence and presence of parthenolide, 1 × 10^6^ cells were washed with PBS by centrifugation for 5 min at 300× *g* and incubated at 37 °C for 15 min with 5 µL of JC-1. Then, the cells were washed in PBS and resuspended in 300 µL of PBS. Cells were analyzed using FC, and the results were presented as a JC-1 monomer/aggregate ratio calculated as the mean fluorescence intensity (MFI) fraction observed for each form.

### 4.5. Oxidative Stress Evaluation/Reactive Oxygen Species Detection

Oxidative stress levels were assessed through the redox imbalance between the ROS levels and antioxidant defenses, namely reduced glutathione (GSH), as previously described [38]. Intracellular peroxides and superoxide anion were measured using the dyes 2,7-dichlorodihydrofluorescein diacetate (DCFH_2_-DA; Molecular Probes, Thermo Fisher Scientific, Waltham, MA, USA) and dihydroethidium (DHE; Molecular Probes, Thermo Fisher Scientific), respectively. A quantity of 1 × 10^6^ untreated and parthenolide-treated cells was incubated with 5 µM of DCFH_2_-DA for 45 min at 37 °C or 5 µM of DHE for 15 min in the dark at RT.

Mercury orange (MO) dye (Sigma-Aldrich) was used to measure the GSH content. Briefly, 1 × 10^6^ cells were incubated with 40 µM of MO for 15 min in the dark at RT. Cells were washed twice with cold PBS by centrifugation at 300× *g* for 5 min and resuspended in the same buffer. Flow cytometry was then performed using a FACSCalibur flow cytometer (Becton Dickinson).

At least 25,000 events were acquired using CellQuest software (Becton Dickinson) and analyzed using Paint-a-Gate (Becton Dickinson). The results were presented using the probe’s mean fluorescence intensity (MFI) in each condition.

### 4.6. pNF-κB Expression by Flow Cytometry

The expression levels of phosphorylated NF-κB p65 were assessed using FC, after intracellular staining performed as described in Section 4.4.3 for activated caspase 3. Briefly, 1 × 10^6^ cells were fixed with 100 μL of fix solution (IntraCell) for 15 min and then washed by centrifugation at 300× *g* for 5 min. Cells were then permeabilized by incubating for 15 min with 100 μL of a permeabilization solution (IntraCell) and a phospho-NF-κB p65 (Ser536) antibody (PE; Cell signaling technology, Danvers, MS, USA). After a washing step, at least 25,000 events were acquired using CellQuest software (Becton Dickinson) and analyzed using Paint-a-Gate (Becton Dickinson). The results are presented as mean fluorescence intensity (MFI) arbitrary units and represent MFI detected in the cells, which is proportional to the protein concentration in each cell.

### 4.7. Gene Expression Analysis

Gene expression levels were determined as previously described [39]. Total RNA from untreated and treated cells was extracted using tripleXtractor (GRiSP, Oporto, Portugal) and reversed transcribed using Xpert cDNA Synthesis Kit (GRiSP). Gene expression levels were performed with real-time quantitative PCR (qPCR) in a QuantStudio™ 5 System (Thermo Fisher Scientific) using Xpert Fast SYBR 2x (GRiSP) and the following primers: *CMYC* F 5′-GTCAAGAGGCGAACACACAAC-3′, *CMYC* R 5′-TTGGACGGACAGGATGTATGC-3′, *TP53* F 5′-CAGCACATGACGGAGGTTGT-3′, *TP53* R 5′-TCATCCAAATACTCCACACGC-3′, *GPX1* F 5′-AGAACGCCAAGAACGAAGA-3′, GPX1 R 5′-TTCACCTCGCACTTCTCG-3′, *TXNRD1* F 5′-TGCGTGTCCTGTGCTTAC-3′, *TXNRD1* R 5′-TGCTGCCTGCCTTCTATTC-3′, *HPRT* F 5′-CCCTGGCGTCGTGATTAGTG-3′, and *HPRT* R 5′-TCGAGCAAGACGTTCAGTCC-3′. Standard curves were created for all studied genes using a serially diluted control sample to assess the reaction efficiency. For each experiment, a non-template control (NTC) was included. The specificity of qPCR reactions was confirmed using the melting curve analysis. Samples were normalized to the endogenous gene (HPRT), and the relative expression values were calculated using the 2^−ΔΔCt^ formula (fold change).

### 4.8. Statistical Analysis

Statistical analysis was performed using the GraphPad Prism 9 software version 9.5.1 for Windows (GraphPad Software, San Diego, CA, USA). A normality test was performed with a Kolmogorov–Smirnov test, and adequate analysis was completed. Student’s *t*-test, analysis of variance, Dunnett’s test, and the Tukey test were used to compare the different groups. A significance level of *p* < 0.050 was considered statistically significant. Results were expressed in mean ± SEM of the number of independent experiments indicated in the figure legends.

## 5. Conclusions

Parthenolide is a very promising natural compound to target lymphoid malignancies. Here, we demonstrated that parthenolide might be considered a possible new therapeutic approach for the treatment of B- and T-lymphoid neoplastic diseases. Importantly, parthenolide significantly induced apoptosis through oxidative stress, with increased reactive oxygen species accompanied by a decrease in reduced glutathione levels. Apoptosis induction was also associated with lower mitochondrial membrane potential, increased activated caspase-3, and FAS-ligand expression, thus confirming an apoptosis-mediated effect. Moreover, the levels of phosphorylated NF-κB were inhibited by parthenolide treatment. Despite these promising results, the mechanism of apoptosis activation by parthenolide is highly dependent on the tumor and cell type. Due to the high heterogeneity of lymphoid malignancies, further in vitro and in vivo studies are needed to further unravel the parthenolide mechanisms in these malignancies. Moreover, the research of new delivery systems might also provide new and improved options for parthenolide application in lymphoid malignancies.

## Figures and Tables

**Table 1:** Effects of parthenolide (PRT) in the cell cycle distribution of lymphoid neoplastic cell lines.

		G_0_/G_1_ (%)	S (%)	G_2_/M (%)
H929 (MM)	Control	54.4 ± 0.9	40.4 ± 1.4	5.2 ± 1.2
	PRT 1 µM	54.0 ± 1.2	40.4 ± 2.3	5.6 ± 1.3
	PRT 5 µM	54.0 ± 1.1	37.0 ± 1.0	9.0 ± 0.7
Farage (DLBCL)	Control	51.8 ± 1.7	37.6 ± 2.0	10.6 ± 0.5
	PRT 1 µM	51.6 ± 1.2	38.6 ± 1.2	9.8 ± 0.3
	PRT 1.5 µM	52.2 ± 1.5	37.2 ± 1.6	10.6 ± 0.5
Raji (BL)	Control	45.7 ± 1.7	42.3 ± 2.0	12.0 ± 1.2
	PRT 1 µM	47.7 ± 2.2	46.0 ± 0.7	9.7 ± 0.8
	PRT 10 µM	37.7 ± 1.8 *	41.7 ± 0.3	20.7 ± 1.7 **
697 (B-ALL)	Control	68.7 ± 5.5	19.7 ± 6.9	11.7 ± 2.9
	PRT 1 µM	72.7 ± 2.3	19.7 ± 6.0	7.7 ± 3.7
	PRT 2.5 µM	72.3 ± 1.3	19.0 ± 5.5	8.7 ± 4.2
KOPN-8 (B-ALL)	Control	69.0 ± 4.4	23.0 ± 4.4	8.0 ± 1.0
	PRT 1 µM	74.7 ± 2.9	18.3 ± 2.8	7.0 ± 1.5
	PRT 2.5 µM	77.7 ± 1.2 **	14.0 ± 1.5	8.3 ± 0.9
CEM (T-ALL)	Control	53.7 ± 2.3	35.0 ± 2.9	11.3 ± 2.3
	PRT 1 µM	51.3 ± 2.4	35.3 ± 2.9	13.3 ± 1.7
	PRT 5 µM	43.7 ± 5.5	41.0 ± 4.3	15.3 ± 1.3
MOLT-4 (T-ALL)	Control	59.3 ± 0.3	35.3 ± 0.8	3.3 ± 1.3
	PRT 1 µM	56.3 ± 2.6	37.3 ± 1.3	3.7 ± 0.7
	PRT 7.5 µM	44.7 ±0.7 **	42.0 ± 0.6 **	13.7 ± 0.8 ***

Data are represented as the percentage of cells in G_0_/G_1_ phase, S phase, and G_2_/M and represent mean ± SEM obtained from at least 3 independent experiments. *, *p* < 0.050; **, *p* < 0.010; ***, *p* < 0.001 compared with untreated cells (control). PRT, parthenolide.

**Table 2:** Oxidative stress parameters induced by parthenolide in lymphoid neoplastic cell lines.

		Peroxides (DCFH2-DA MFI)	Superoxide Anion (DHE MFI)	Reduced Glutathione (MO MFI)
H929 (MM)	Control	100.0 ± 0.0	100.0 ± 0.0	100.0 ± 0.0
	PRT 1 µM	112.5 ± 9.4	134.3 ± 11.6	80.3 ± 4.8
	PRT 5 µM	139.9 ± 20.5	131.5 ± 15.0	74.4 ± 3.1 *
Farage (DLBCL)	Control	100.0 ± 0.0	100.0 ± 0.0	100.0 ± 0.0
	PRT 1 µM	83.9 ± 9.2	114.8 ± 7.7	78.7 ± 2.7 **
	PRT 1.5 µM	105.1 ± 12.7	123.0 ± 7.6	80.0 ± 6.3 *
Raji (BL)	Control	100.0 ± 0.0	100.0 ± 0.0	100.0 ± 0.0
	PRT 1 µM	101.5 ± 3.6	105.3 ± 2.8	69.2 ± 7.3 ***
	PRT 10 µM	107.6 ± 1.6 *	133.0 ± 9.9 *	23.7 ± 1.4 ***
697 (B-ALL)	Control	100.0 ± 0.0	100.0 ± 0.0	100.0 ± 0.0
	PRT 1 µM	107.2 ± 1.9	104.7 ± 1.3	84.5 ± 8.5
	PRT 2.5 µM	142.7 ± 18.4 *	112.3 ± 3.4 **	74.2 ± 5.0 *
KOPN-8 (B-ALL)	Control	100.0 ± 0.0	100.0 ± 0.0	100.0 ± 0.0
	PRT 1 µM	109.4 ± 2.3	123.1 ± 7.9	97.0 ± 2.4
	PRT 2.5 µM	125.5 ± 7.4 **	148.3 ± 17.8 *	71.1 ± 5.6 ***
CEM (T-ALL)	Control	100.0 ± 0.0	100.0 ± 0.0	100.0 ± 0.0
	PRT 1 µM	105.6 ± 2.5 *	103.4 ± 1.3	90.4 ± 3.4
	PRT 5 µM	117.1 ± 1.4 ***	112.4 ± 1.4 ***	75.3 ± 4.9 ***
MOLT-4 (T-AL0 /2L)	Control	100.0 ± 0.0	100.0 ± 0.0	100.0 ± 0.0
	PRT 1 µM	120.8 ± 10.9	104.5 ± 2.4	68.4 ± 6.6 **
	PRT 7.5 µM	170.9 ± 11.8 ***	139.6 ± 7.3 ***	52.3 ± 9.3 ***

Data are represented as mean fluorescence intensity (MFI) normalized to control and represent mean ± SEM obtained from at least 3 independent experiments. *, *p* < 0.05; **, *p* < 0.01; ***, *p* < 0.001 compared with untreated cells (control). DCFH2-DA, 2,7-dichlorodihydrofluorescein diacetate; DHE, dihydroethidium; MO, mercury orange; PRT, parthenolide.

## Data Availability

All data generated or analyzed during this study were included in this published article.
